# Reduced protective efficacy of hepatitis B vaccine among fully vaccinated children in Ethiopia

**DOI:** 10.1371/journal.pone.0288355

**Published:** 2023-07-07

**Authors:** Adane Adugna, Gebereselassie Demeke, Milkiyas Toru, Dareskedar Tsehay, Ahmed Esmael, Adane Mihret, Andargachew Mulu

**Affiliations:** 1 Medical Laboratory Science, College of Health Sciences, Debre Markos University, Debre Markos, Ethiopia; 2 Armauer Hansen Research Institute, Addis Ababa, Ethiopia; Animal Health Institute, Ethiopia, ETHIOPIA

## Abstract

**Background:**

Hepatitis B vaccination is recommended for all children at birth within 24 hours or during childhood.

**Objective:**

This study was aimed to evaluate protective efficacy of hepatitis B vaccine and estimate the sero-prevalence of hepatitis B virus infection among vaccinated children.

**Materials and methods:**

A community-based cross-sectional study was conducted from March, 2021 to October, 2021 in Debre Markos town. A simple random sampling technique was used to select 165 fully vaccinated children aged 5–12 years old. A serum sample was used to determine hepatitis B surface antigen (HBsAg), anti-hepatitis B core antibody (anti-HBc), anti-hepatitis B surface antibody titer (anti-HBs) using ELISA.

**Results:**

The seroprevalence of HBsAg and anti-HBc anti-body was found to be 4.2% and 4.8% respectively. Of 165 fully vaccinated children, 129 (78.2%) had anti-HBs titer ≥ 10 mIU/ml. Among 129 sero-protected children, 76 (58.9%) were hypo-responders whereas the rest 53 (41.1%) were good responders. Those children within the age group of 5–7 years were 2.9 times (AOR: 2.873, 95% CI: 1.156, 7.141) (P<0.023) more likely to respond to HBV vaccine. Multivariate logistic regression revealed that children who were born from HBV positive mothers (AOR 3.917, 95% CI: 1.456, 5.365, P<0.027) and those who had history of injectable medications (AOR 9.232, 95% CI: 1.503, 11.697, P<0.016) were more likely to be HBsAg positive. Children who had history of hospital admission (AOR 6.973, 95% CI: 1.495, 8.530, P<0.013) were more likely to be anti-HBcAb positive.

**Conclusions:**

There was an intermediate prevalence of childhood HBV infection despite being vaccinated suggesting low protective efficacy of hepatitis B vaccine in the study area.

## Introduction

According to the World Health Organization, one-third of the global population has serological markers of active HBV infection. More than 350 million are chronic carriers with HBV [1–3] and 1.2 million die annually from liver cancer [4, 5]. Africa has the second biggest quantity of persistent HBV carriers next to Asia and considered as a region of extraordinarily endemicity [6]. With 60 million people who are chronically infected due to perinatal transmission [7, 8]. Vertical transmission to neonates results in persistent infection in more than 90% of children [9]. Babies born from mothers with HBsAg and HBeAg markers have 90% a probability of infection [10], and 15 to 25% may die from hepatocellular carcinoma in their life time [11]. In Ethiopia according to the previous seroepidemiological survey of Addis Ababa, 7% of individuals had HBsAg, and 70% had at least one HBV marker [12].

Nowadays, about 194 countries have performed HBV vaccination program for infants [13]. After complete vaccination the non-responder, hypo-responder, and good- responder may exist with an anti-HBs titer less than 10 mIU/ml, between 10–100 mIU/ml, and greater than100 mIU/ml respectively [14, 15]. In Ethiopia, HBV vaccination is part of the pentavalent [DPT-HiB-HepB] vaccine which has been introduced to the national Expanded Program for Immunization (EPI) in 2007. According to EPI schedules, HBV vaccine is given at 6, 10, and 14 weeks of age after delivery [16]. However, the vaccine is not always protective against HBV due to different reasons including viral, host immune and genetics, socio demographic, cultural and programmatic factors [17, 18].

Due to those factors, 15–50% of the vaccinated children may have undetetctable concentrations of anti-hepatitis B surface antibody after 5–15 years of vaccination and 10% still could be susceptible to HBV infection [19, 20]. In Ethiopia, pentavalent vaccine coverage is increasing and has reached to 90%. However, studies still show susceptibility to HBV infection despite high vaccine coverage [21]. There is limited data on seroprevalence and vaccine efficacy among fully vaccinated children in Ethiopia. Thus; this study aimed to estimate seroprevalence of HBV infection, evaluate the protective efficacy of HB vaccine, and identify associated factors for reduced vaccine efficacy among fully vaccinated children in Debre Markos town, Northwest Ethiopia.

## Materials and methods

### Study design, period and setting

A community-based cross-sectional study was conducted among hepatitis B vaccinated children in Debre Markos town, Amhara regional state, Northwest Ethiopia, from March 2021 to October 2021. Based on the report from East Gojjam Zone administrative office, the number of children < 15 years was 18,345 of which the number of girls and boys were 10,020 and 8,325 respectively.

### Inclusion an exclusion criteria

All hepatitis B fully vaccinated children whose ages were from 5–12 years and live in Debre Markos town were considered as source population and those hepatitis B fully vaccinated children whose ages were from 5–12 years, who fulfilled

eligibility criteria and were found at home during the data collection period were taken as study population. All hepatitis B vaccinated children who volunteered to participate in the study, whose parents or legal guardians volunteered to give assent and those children who took full dose of hepatitis B vaccine [as evidenced by showing vaccination card] were included in the study. Those study participants who did not complete the full dose of the hepatitis B vaccine, who or whose parents were severely sick (as unable to give assent on behalf of their children) during the data collection period and could not give blood samples were excluded from the study.

### Sample size determination and sampling techniques

The sample size was calculated using single population proportion formula by taking 89% (0.89) of the prevalence in vaccine response derived from a study done in Addis Ababa, Ethiopia [22]. By considering a prevalence of 89%, 5% margin of error, 95% confidence interval and 10% non-response rate

$n=\frac{{\left(\frac{Za}{2}\right)}^{2}pq}{d^{2}}$ n = (1.96)^2^/ (0.05)^2^ *0.89*0.11 = 150

Where

n = sample

Z = 1.96 at 95% C.I

p = prevalence = 0.89

q = 1-p = 1–0.89 = 0.11

d = margin of error = 0.05

Non-response rate = 10% of 150 = 10%*150 = 10 ∕ 100*150 = 15

Therefore, the final sample size (n) was calculated as n = 150+15 = 165.

Simple random sampling technique was used to recruit the study participants from March, 2021 to October, 2021 until the required sample size was obtained among hepatitis B vaccinated children at the selected kebeles in Debre Markos town. Debre Markos Town has 11 kebeles out of them 5 kebeles (45%) were selected by lottery method so as to keep representativeness. Then total sample size was allocated proportionally to each kebeles depending on the total number of vaccinated children from 5 to 12 years of age. One child was selected randomly (lottery method) from those households having two and more children.

### Data collection procedures

After the participant’s family agreed to take part in the study and signed on informed written assent form, socio-demographic characteristics and associated factors data were collected by using a pretested semi-structured questionnaire. Each child’s weight and height were measured and body mass index was calculated in percentile. Authors were blind and had no information about the study participants during or after data collection.

### Laboratory procedures

Five ml of venous blood was aseptically collected from each study participant from March, 3/25/2021 to October, 10/5/2021 and transported to Debre Markos blood bank laboratory within one hour. Serum was then separated by centrifugation, and stored at -20°C until transported to Armauer Hansen Research Institute (AHRI) for analysis. Serum level of HBsAg, anti-HBcAb, and anti-HBs were determined using sandwich Enzyme-Linked Immunosorbent Assay (ELISA), where antigens/monoclonal antibodies were used both for capture and detection [BIO-RAD, Monolisa, France]. For the determination of anti-HBs level, quantitative type of sandwich ELISA was used. The test kits have a clamed sensitivity of 100% and specificity of 99.28%. Each test procedure was performed according to the manufacturer’s instruction [https://www.bio-rad.com/].

### Data processing and analysis

After checking the data completeness, data were entered into- Epidata software version 3.1 and exported to SPSS software version 25 for analysis. The results were presented as tables and figure. Binary logistic regression was used to show the association between dependent and independent variables. In bivariate logistic regression, a p-value <0.25 were candidates for multivariate logistic regression. Multivariate logistic regression was used in terms of adjusted odds ratio (AOR) with 95% confidence intervals and a p-value < 0.05 was considered statistically significant.

#### Ethical consideration and consent to participate

This study was ethically approved by the research and ethical committee of College of Health Science, Debre Markos University with reference number (HSC/R/C/Ser/PG/CO/277/12/13). It was performed in accordance with the relevant guidelines and regulations. Written informed assent was taken from the participant’s guardian and consent was obtained from all participants.

## Results

### Socio-demographic characteristics

A total of 165 children with the age range of 5–12 years participated in this study. The median age of the study participants was 8.00 ± 2.58. About 45.5% of the study participants were found within the age group of 5–7 years and the majority of them 89/165 (53.9%) were males. One hundred and fifty (90.9%) of participants were from urban area (Table 1).

**Table 1:** Socio–demographic characteristics of children and their parents at Debre Markos town, North West Ethiopia in 2021.

Socio-demographic variables	Age category	Frequency	Percent
Age category	5–7	75	45.5
	8–10	49	29.7
	≥11	41	24.8
Sex of child	Male	89	53.9
	Female	76	46.1
Birth place of child	Rural	15	9.1
	Urban	150	90.9
Educational status of parents	Illiterate	50	30.3
	Primary school	20	12.1
	Secondary school	40	24.2
	College and above	55	33.3
Occupation of parents	Farmer	21	12.7
	Government employer	43	26.1
	Private	24	14.5
	Daily laborer	17	10.3
	House wife	29	17.6
	Merchant	31	18.8
Total		165	100.0

### Sero-positivity of HBsAg and anti-HBc

The overall positivity of HBsAg among all study participants was 7/165 (4.2%) (95% CI: 1.0–5.0). Of seven children who were positive for HBsAg, 6/7 (85.7%) of them were males. Furthermore, the prevalence of HBsAg positivity in 5–7 years children was 5/7 (71.4%). The overall prevalence of anti-HBc antibodies among the study participants was 8/165 (4.8%) (95% CI: 2.0–6.0). From the total prevalence of anti-HBc antibodies, 4/8 (50.0%) was from female participants. About 4/8 (50.0) % anti-HBc antibody prevalence was from children whose ages were found within the age group of ≥ 11 years (Table 2).

**Table 2:** Sero–positivity of HBsAg and anti–HBc with socio–demographic variables.

Variables	Category	HBsAg status			Anti-HBc Status			Anti-HBs titers (mIU/ml)		
		Positive	Negative	Total	Positive	Negative	Total	<10	≥10	Total
Age	5–7	5(71.4%)	70(44.3%)	75	2(25.0%)	73(46.5%)	75	11(30.6%)	64(49.6%)	75
	8–10	2(28.6%)	47(29.7%)	49	2(25.0%)	47(29.9%)	49	11(30.5%)	38(29.5%)	49
	≥11	0(0.0%)	41(25.9%)	41	4(50.0%)	37(236%)	41	14(38.9%)	27(20.9%)	41
Sex	Male	6(85.7%)	83(52.5%)	89	4(50.0%)	85(54.1%)	89	20(55.6%)	69(53.5%)	89
	Female	1(14.3%)	75(47.5%)	76	4(50.0%)	72(45.8%)	76	16(44.4%)	60(46.5%)	76
Educational status of parents	Illiterate	3(42.8%)	47(29.7%)	50	2(25.0%)	48(30.6%)	50	15(41.7%)	35(27.1%)	50
	Primary	1(14.3%)	19(12.0%)	20	2(25.0%)	18(11.5%)	20	3(8.3%)	17(13.2%)	20
	Secondary	1(14.3%)	39(24.7%)	40	2(25.0%)	38(24.2.0)	40	9(25.0%)	31(24.0%)	40
	College and above	2(28.6%)	53(33.5%)	55	2(25.0%)	53(33.7%)	55	9(25.0%)	46(35.7%)	55
Occupation of parents	Farmer	1(14.3%)	20(12.6%)	21	2(25.0%)	19(12.1%)	21	4(11.1%)	17(13.2%)	21
	Government employer	1(14.3%)	42(26.6%)	43	0(0.0%)	43(27.4%)	43	10(27.8%)	33(25.6%)	43
	Private	1(14.3%)	23(14.5%)	24	2(25.0%)	22(14.0%)	24	3(8.3%)	21(16.3%)	24
	Daily laborer	2(28.5%)	15(9.5%)	17	1(12.5%)	16(10.2%)	17	7(19.4%)	10(7.8%)	17
	House wife	1(14.3%)	28(17.7%)	29	1(12.5%)	28(17.8%)	29	7(19.4%)	22(17.0%)	29
	Merchant	1(14.3%)	30(19.0%)	31	2(25.0%)	29(18.5%)	31	5(13.9%)	26(20.1%)	31

### Distribution of HBsAg and anti-HBcAb

Of the total prevalence of HBsAg, about 14.3% was from children who were born from mothers with previous history of HBV infection. The prevalence of HBsAg among obese children was 2/7(28.6%). The prevalence of anti-HBc antibody among children with previous history of injectable medication was 1/8(12.5%) (Table 3).

**Table 3:** Distribution of HBsAg, anti–HBcAb and anti–HBs with clinical variables.

Clinical or cultural variables	Category	HBsAg status			Anti-HBc status			Anti-HBs titers (mIU/ml)		
		Positive	Negative	Total	Positive	Negative	Total	<10	≥10	Total
Full dose of HBV vaccine	Yes	7(100.0%)	158(100.0%)	165	8(100.0%)	157(100.0%)	165	36(100.0%)	129(100.0%)	165
	No	0(0.0%)	0(0.0%)	0	0(0.0%)	0(0.0%)	0	0(0.0%)	0(0.0%)	0
Mother’s HBV status	Yes	1(14.3%)	2(1.3%)	3	1(12.5%)	2(1.3%)	3	0(0.0%)	3(2.3%)	3
	No	6(85.7%)	156(98.7%)	162	7(87.5%)	155(98.7%)	162	36(100.0%)	126(97.7%)	162
BMI status in percentile	Normal	3(42.8%)	125(79.1%)	128	8(100.0%)	120(76.4%)	128	34(94.4%)	94(72.9%)	128
	Uw	2(28.6%)	16(10.1%)	18	0(0.0%)	18(11.5%)	18	0(0.0%)	18(14.0%)	18
	Ow	0(0.0%)	12(7.6%)	12	0(0.0%)	12(7.6%)	12	2(5.6%)	10(7.7%)	12
	Obese	2(28.6%)	5(3.2%)	7	0(0.0%)	7(4.5%)	7	0(0.0%)	7(5.4%)	7
Smoking	Yes	0(0.0%)	0(0.0%)	0	0(0.0%)	0(0.0%)	0	0(0.0%)	0(0.0%)	0
	No	7(100.0%)	158(100.0%)	165	8(100.0%)	157(100.0%)	165	36(100.0%)	129(100.0%)	165
Hospital admission	Yes	2(28.6%)	19(12.0%)	21	4(50.0%)	17(10.8%)	21	4(11.1%)	17(13.2%)	21
	No	5(71.4%)	139(88.0%)	144	4(50.0%)	140(89.2%)	144	32(88.9%)	112(86.8%)	144
Blood transfusion	Yes	1(14.3%)	2(1.3%)	3	1(12.5%)	2(1.3%)	3	1(2.8%)	2(1.6%)	3
	No	6(85.7%)	156(98.7%)	162	7(87.5%)	155(98.7%)	162	35(97.2%)	127(98.4%)	162
Injectable medications	Yes	3(42.9%)	12(7.6%)	15	1(12.5%)	14(8.9%)	15	4(11.1%)	11(8.5%)	15
	No	4(57.1%)	146(92.4%)	150	7(87.5%)	143(91. 0%)	150	32(88.9%)	118(91.5%)	150
Circumcision	Yes	5(71.4%)	69(43.7%)	74	4(50.0%)	70(44.6%)	74	14(38.9%)	60(46.5%)	74
	No	2(28.6%)	89(56.3%)	91	4(50.0%)	87(55.4%)	91	22(61.1%)	69(53.5%)	91
Body piercing	Yes	3(42.9%)	20(12.7%)	23	1(12.5%)	22(14.0%)	23	8(22.2%)	15(11.6%)	23
	No	4(57.1%)	138(87.3%)	142	7(87.5%)	135(86.0%)	142	28(77.8%)	114(88.4%)	142
Tattooing	Yes	0(0.0%)	12(7.6%)	12	0(0.0%)	12(7.6%)	12	6(16.7%)	6(4.7%)	12
	No	7(100.0%)	146(92.4%)	153	8(100.0%)	145(92.4%)	153	30(83.3%)	123(95.3%)	153

NB: % = percentile

BMI = Body Mass Index

Normal weight BMI = 5th–85th %ile

Underweight (Uw) = <5th %ile

Overweight (Ow) = 86 th–95th %ile

Obese = ≥96th %ile

### Seroprotection level of HBV vaccine

Among vaccinated children, 129/165 (78.2%) (95% CI: 72.0–85.0%) of them had anti-HBs titer ≥ 10 mIU/ml. Out of this, 76 (58.9%) were hypo-responders (with anti-HBs titer of 10–100 mIU/ml) whereas the rest 53(41.1%) were good responders (with anti-HBs titer of >100).

### Factors associated with HBsAg and anti-HBcAb positivity

In multivariable logistic regression, children born to mothers with HBV infection were 3.9 times (AOR: 3.917, 95% CI: 1.456, 5.365) (P<0.027) more likely to be HBsAg seropositive as compared to those who were born to mothers without previous history of HBV infection. Children who had previous history of injectable medications were 9 times (AOR: 9.232, 95% CI: 1.503, 11.697) (P<0.016) more likely to be HBsAg positive than those without previous history of injectable medications (Table 4). Moreover, children who had previous history of hospital admission were 6.9 times (AOR: 6.973, 95% CI: 1.495, 8.530) (P<0.013) more likely to be anti-HBc antibody positive as compared to their counterparts.

**Table 4:** Bivariate and multivariate logistic regression of associated factors for HBsAg p positivity at Debre Markos town, North West Ethiopia in 2021.

Variables	Category	HBsAg status					
		Positive	Negative	COR(95%CI)	P-V	AOR(95%CI)	P-V
Mother’s HBV status	Yes	1(14.3%)	2(1.3%)	13.000(1.030,16.040)	0.047*	3.917(1.456,5.365)	0.027**
	No	6(85.7%)	156(98.7%)	Ref		Ref	
Hospital admission	Yes	2(28.6%)	19(12.0%)	2.926(0.530,16.155)	0.218*	1.539(0.185,12.828)	0.690
	No	5(71.4%)	139(88.0%)	Ref		Ref	
Blood transfusion	Yes	1(14.3%)	2(1.3%)	13.000(1.030,16.040)	0.047*	4.035(0.161,10.900)	0.396
	No	6(85.7%)	156(98.7%)	Ref		Ref	0.396
Injectable medication	Yes	3(42.9%)	12(7.6%)	9.125(1.827,45.582)	0.007*	9.232(1.503,56.697)	0.016**
	No	4(57.1%)	146(92.4%)	Ref		Ref	
Body Piercing	Yes	3(42.9%)	20(12.7%)	5.175(1.078,24.842)	0.040*	4.264(0.640,28.405)	0.134
	No	4(57.1%)	138(87.3%)	Ref		Ref	

NB: *: Candidate variable for multivariate analysis at P < 0.25 and **: significant variable by the multivariate analysis at P < 0.05

COR: crude odds ratio

AOR: adjusted odds ratio

CI: confidence interval

P–V: p–value

Ref: reference

### Factor associated with seroprotection of HBV vaccine

In multivariate logistic regression, children who were found within the age group of 5–7 years were 2.9 times (AOR: 2.873, 95% CI: 1.156, 7.141) (P<0.023) more likely to respond to HBV vaccine as compared to those whose ages were > 7 years.

## Discussion

The overall sero-positivity of HBsAg among vaccinated children from 5–12 years was 4.2% (95% CI: 1.0–5.0). This result tells us the study area is found under the category of intermediate endemicity for HBV infection [23]. This finding is consistent with similar studies conducted in Hawassa (4.4%), Gondar (4.2%), Jimma (2.1%), and Harar (1.1%), [24–27]. It is also comparable with other studies conducted in low and high income countries [28–35]. However, the seroprevalence of HBsAg in the present study is relatively higher than the results reported among vaccinated children from Addis Ababa (0.4%) [36], Uzbekistan-Japan (0.8%) [37], Iran (0.6%) [38], Italy (0.6%) [39], Egypt (0.6%) [40], and Dakahleya-Egypt (0.4%) [41] and lower than the result reported from china (20.49%) [42]. These variation may be due to difference in host genetic factors, ethnicity, cultural practice, socioeconomic status, family history of HBV and structural difference including clinical practice, immunization coverage of HBV vaccine, inclusion of birth dose of HBV vaccine within 24 hours, and type of laboratory method used for HBsAg detection. According to our study, males were more susceptible to HBV infection (6.7 vs 1.3%) than females.

Previous history of child’s mother for HBV infection and child’s injectable medications were identified as important determinants for HBV infection. This is in accordance with the findings reported from Hawassa [24]. This might be due to vertical transmission of HBV infection for those children born to HBV infected mothers and there was unsafe utilization of medical equipment during therapeutic injections [43, 44].

The overall seroprevalence of anti-HBc positivity in this study was 4.8% (95% CI: 2.0–6.0) which is comparable with previous results reported from Addis Ababa (5.6%) [36] and Gondar (6.3%) [25] and other similar studies elsewhere [31, 34, 38, 45, 46]. On the other hand, the current finding of anti-HBc seroprevalence is relatively higher than the previous findings reported from Jimma (1.1%) [26], Niakhar-Senegal (1.5%) [35], and Dakahleya-Egypt (0.11%) [41]. In contrast, higher prevalence were reported from Hawassa city (19.5%) [24] and other country; Gambian Villages (10.2%) [33]. This variations may be difference in; geographical area, age range of study participants, vaccination rate of HBV vaccine, income level, socio cultural practices, and inclusion of birth dose of HBV vaccine within 24 hours. In this study, children who had history of hospital admission were more likely to be anti-HBc antibody positive. This might be due to the horizontal transmission of the virus from the hospital environment.

The seroprotection level of hepatitis B vaccine among 5–12 years children in this study was 78.2% (95% CI: 72.0–85.0%) which is low since safe and effective HBV vaccine has a protection level of 98 to100% according to WHO report [47]. This figure is in line with results reported from Italy (79.2%) [38] and Yemen (72.2%) [48], and relatively lower than studies conducted in China (88.3%) [45], Cameroon (97.5%) [49], and Senegal (87%) [50] and higher than the findings reported from India (68%) [51], Burkina Faso (61.6%) [52], Egypt (57.7%) [41], and Ethiopia which ranges from 32.2–58.4% [25, 36, 53]. The disparity might be due to; differences in cold chain system of the vaccine, differences in nutritional status, age groups, and different extent of exposure to natural boosters.

The protective levels of HBV vaccine in this study seemed higher among females than males (53.5 vs 46.5%) though not statistically significant and is in agreement with the study conducted in Yemen (75.7% vs 68.4%) [48], and Addis Abeba (54.7% vs 53.9%) [36] and could be related to a stronger innate, humoral, and cell-mediated immune responses to viral vaccines in females than males [54].The protective levels of HBV vaccine (≥10 mIU/ml anti-HBs antibody) were decreased from 49.6 to 29.5.6 to 20.9% as the age of the child increased from age group 5–7 to 8–10 and ≥ 11 years respectively which is similar with figures reported from Kurdistan Region-Iraq [55], and Addis Ababa [36]. These differences in the age groups were statistically significant in the present study and children who were found within the age group of 5–7 years were 2.9 times more likely to respond to HBV vaccine as compared to those whose ages were in 8–10 and ≥ 11 years and this is due to the concentration of vaccine-induced anti-HBs levels in the vaccinated children will decline as the time of vaccination increases [56].

In conclusions, the seroprevalence of hepatitis B infection was intermediate among vaccinated children in the study area. Mother’s HBV status was significantly associated with HBsAg positivity. There should be regular early screening of vaccinated children for HBV infection. The protective efficacy of HBV vaccine in the present study was low. Thus, determination of levels of anti-HBs antibodies should be done, and then booster doses must be given for non-responders to increase the concentration of anti-HBs levels and prevent waning immunity.

Limitations of the study: Even though this study has paramount public health importance, it was done with small sample size. As a result, it is difficult to infer seroprevalence of HBV infection and seroprotection of HBV vaccine to the entire population with this sample size.

## Supporting information

S1 Data (SAV)
